# Tuberculosis in Dr Granville's mummy: a molecular re-examination of the earliest known Egyptian mummy to be scientifically examined and given a medical diagnosis

**DOI:** 10.1098/rspb.2009.1484

**Published:** 2009-09-30

**Authors:** Helen D. Donoghue, Oona Y.-C. Lee, David E. Minnikin, Gurdyal S. Besra, John H. Taylor, Mark Spigelman

**Affiliations:** 1 Centre for Infectious Diseases and International Health, University College London, London W1T 4JF, UK; 2 School of Biosciences, University of Birmingham, Edgbaston, Birmingham B15 2TT, UK; 3 Department of Ancient Egypt and Sudan, The British Museum, Great Russell Street, London WC1B 3DG, UK; 4 Kuvin Centre for the Study of Infectious and Tropical Diseases, The Hebrew University, Hadassah Medical School, PO Box 12272, Jerusalem 91120, Israel

**Keywords:** ancient DNA, ancient Egypt, Granville mummy, high-performance liquid chromatography, *Mycobacterium tuberculosis*, mycolic acid

## Abstract

‘Dr Granville's mummy’ was described to the Royal Society of London in 1825 and was the first ancient Egyptian mummy to be subjected to a scientific autopsy. The remains are those of a woman, Irtyersenu, aged about 50, from the necropolis of Thebes and dated to about 600 BC. Augustus Bozzi Granville (1783–1872), an eminent physician and obstetrician, described many organs still *in situ* and attributed the cause of death to a tumour of the ovary. However, subsequent histological investigations indicate that the tumour is a benign cystadenoma. Histology of the lungs demonstrated a potentially fatal pulmonary exudate and earlier studies attempted to associate this with particular disease conditions. Palaeopathology and ancient DNA analyses show that tuberculosis was widespread in ancient Egypt, so a systematic search for tuberculosis was made, using specific DNA and lipid biomarker analyses. Clear evidence for *Mycobacterium tuberculosis* complex DNA was obtained in lung tissue and gall bladder samples, based on nested PCR of the IS*6110* locus. Lung and femurs were positive for specific *M. tuberculosis* complex cell-wall mycolic acids, demonstrated by high-performance liquid chromatography of pyrenebutyric acid–pentafluorobenzyl mycolates. Therefore, tuberculosis is likely to have been the major cause of death of Irtyersenu.

## 1. Introduction

**Figure 1.**
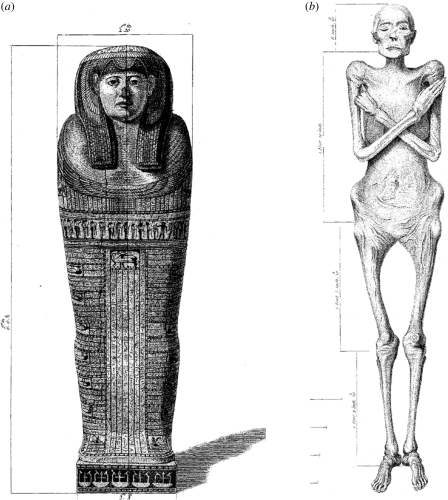
The original appearance of the Granville mummy. (*a*) Inner coffin lid; (*b*) unwrapped mummy (Granville 1825).

The presentation made by Augustus Bozzi Granville to the Fellows of the Royal Society (Granville 1825) was the culmination of a study that had aroused great public interest and laid the foundations of the scientific study of ancient Egyptian mummies. Earlier descriptions of mummies, attempts to describe the wrappings and materials used in mummification and the exterior state of the human remains, were fully reviewed by Granville. However, he was fortunate to gain access to a particularly well-preserved specimen (figure 1*a*,*b*) and had the prerequisite medical knowledge to take full advantage of this opportunity. The mummy, from the necropolis of Thebes, was of the lady Irtyersenu, of the 26th Dynasty, dated to *ca* 600 BC (Hedges *et al*. 1997). After detailed observations of the wrappings and the exterior features of the mummy, Granville decided to perform a detailed (but destructive) autopsy. As he stated (Granville 1825, p. 281):

Having proceeded thus far into my inquiry into the state of preservation of the mummy before me, I determined, perfect and beautiful as it was, to make it the object of further research by subjecting it to the anatomical knife, and thus to sacrifice a most complete specimen of the art of Egyptian embalming, in hopes of eliciting some new facts illustrative of so curious and interesting a subject;

Granville, who was an eminent physician and obstetrician, observed that the mummy was that of a woman aged around 50, who had been corpulent. She showed signs of what we now recognize as osteoporosis, but of particular interest was a mass around the right ovary, which Granville described as ‘ovarian dropsy’ or cancer and led to his conclusion that this was the cause of death. However, more recent studies (Strouhal 1976; Nunn & Tapp 2000) suggest that this was a benign cystadenoma of the ovary, which would not have been fatal.

The residual organs and bones of the Granville mummy were displayed in a wooden cabinet, which is now in the British Museum in London. Further studies were carried out in 1994 and subsequent years, which are described in a book due to be published by the British Museum Press (Harer & Taylor in press). The histological studies of the mummy tissues mentioned above noted a potentially fatal pathological condition—a pulmonary exudate (Tapp in press). In an attempt to determine the cause of death, further investigations were carried out. In one of these, tissue from the Granville mummy was tested using the ParaSight-*F*-test for *Plasmodium falciparum* histidine-rich protein-2. A positive finding suggested that the lady Irtyersenu was suffering from untreated *P. falciparum* malaria when she died (Miller *et al*. 1994). However, it was subsequently discovered that this test gives false-positive reactions in the presence of serum rheumatoid factor (Laferl *et al*. 1997; Bianucci *et al*. 2008). During the early 1990s, several attempts were made to detect microbial or human DNA from the Granville mummy by PCR. These remain unpublished as negative results were obtained and investigators concluded that the extracts were strongly inhibitory to DNA amplification. As part of a broader study on historical and ancient malaria, a subsequent attempt to detect *P. falciparum* DNA from the Granville mummy was inconclusive (Taylor *et al*. 1997).

Tuberculosis has long been recognized in Egyptian mummies by palaeopathological changes, such as Pott's disease, and *Mycobacterium tuberculosis* complex DNA has been detected and characterized from the Predynastic era (Crubézy *et al*. 1998), the Old, Middle and New Kingdoms (Nerlich *et al*. 1997; Zink *et al*. 2003*a*). Therefore, the aim of this study was to determine whether the lady Irtyersenu had evidence of tuberculosis in her tissues, which may have been her cause of death. Because of the reported difficulty of obtaining amplifiable DNA from this mummy, an additional method was used: the direct detection of specific cell-wall mycolic acid biomarkers of the *M. tuberculosis* complex, using high-performance liquid chromatography (HPLC). This has been used previously to confirm cases of tuberculosis indicated by *M. tuberculosis* complex DNA (Donoghue *et al*. 1998; Gernaey *et al*. 2001) and the technique has recently been refined and increased in sensitivity, thereby permitting the direct detection and quantification of *M. tuberculosis* complex mycolic acid derivatives in human remains 9000 years old (Hershkovitz *et al*. 2008).

## 2. Material and methods

### (a) Samples

Samples taken in the early 1990s were available—lung tissue, and bone from the left and right femurs and the hand of the Granville mummy. In addition, additional samples were collected from the lung, gall bladder and soft tissues that may be of pleura or diaphragm. Samples were examined independently at UCL (DNA analysis) and University of Birmingham (cell-wall mycolic acid biomarkers).

### (b) Precautions against contamination

The recommended protocols of ancient DNA (aDNA) work (O’Rourke *et al*. 2000; Donoghue & Spigelman 2006; Taylor *et al*. in press) were followed. In brief, samples were processed in laboratories where no work with modern *M. tuberculosis* has ever been performed. There was strict separation of work areas between DNA extraction, PCR set-up, nested and post-PCR procedures using different laboratories, demarcated by labels, colour of laboratory coats and backed up by staff training. Within laboratories, strict guidelines were followed regarding cleaning and non-sharing of equipment. Dedicated sets of pipettes were used for PCR set-up and were stripped and cleaned in detergent and ethanol before each experiment. Fresh sterile filter tips were used routinely. Surfaces and equipment in contact with sample tubes (centrifuges, rotors, mixers, etc.) were cleaned before each assay and use of sterile and pre-purchased reagents minimized contamination risk.

### (c) DNA extraction

DNA extraction procedures have been described previously (Donoghue *et al*. 2005). Three separate extractions were performed (electronic supplementary material, table S1). In brief, approximately 25 mg of sample was pre-incubated in Proteinase K/EDTA at 56°C for 24–72 h. Owing to the reported difficulty of obtaining amplifiable DNA from this material, an aliquot from each sample was treated with 0.1 M *N*-phenacylthiazolium bromide, a reagent that cleaves glucose-derived protein cross-links (Poinar *et al*. 1998). Thereafter, all samples were lysed in guanidium thiocyanate solution at 56°C (Boom *et al*. 1990) and vigorously mixed with small glass beads to release any mycobacterial DNA. DNA was captured onto silica by using spin columns (DNeasy tissue kit, Qiagen Ltd). After washing and drying, DNA was eluted from the silica, aliquoted and used immediately or stored at −20°C. Negative extraction controls were always included.

### (d) DNA amplification and detection

The *M. tuberculosis* complex was detected by targeting a specific region of the repetitive element IS*6110* (Eisenach *et al*. 1990; Hellyer *et al*. 1996). A two-tube nested PCR was used, which yielded an outer product of 123 bp and a nested PCR product of 92 bp (Spigelman & Lemma 1993; Taylor *et al*. 1996). The PCR mix included 10 mM bovine serum albumin to reduce PCR inhibition (Forbes & Hicks 1996; Abu Al-Soud & Rådström 2000). A 50 µl reaction mix was used with 5 µl of DNA extract. Tenfold dilutions of DNA extract were also tested to reduce any inhibition. In a parallel set of experiments, 1 M betaine was included in the PCR mix as a facilitator (Weissensteiner & Lanchbury 1996). A hot-start *Taq* polymerase was used to minimize non-specific primer and template binding. Negative controls were always included. PCR product was detected by staining with ethidium bromide and visualized under ultraviolet light. In order to characterize the predominant DNA molecule, direct sequencing of the whole PCR amplicon was performed by MWG-BIOTECH AG (Ebersberg, Germany).

### (e) Mycolic acid derivatization and high-performance liquid chromatography analysis

Extracts of a stored lung tissue sample taken previously (sample 1: 29.6 mg), a new lung sample (sample 2: 69.9 mg) and combined left and right femurs (71.2 mg) were analysed by HPLC for mycolic acids. The detailed protocols have been described previously (Hershkovitz *et al*. 2008). In brief, samples were hydrolysed and long-chain fatty acids derivatized to pentafluorobenzyl (PFB) esters by established procedures (Minnikin *et al*. 1993). PFB mycolate fractions were isolated using normal phase cartridges and reacted with pyrenebutyric acid (PBA) to give PBA–PFB-derivatized mycolates, which, after standard clean-up procedures, were examined by reverse-phase HPLC for profiles similar to standard *M. tuberculosis*. It has been established that modern tuberculosis can be diagnosed by recognition of a very characteristic reverse-phase HPLC profile of mycolic acids, shown by members of the *M. tuberculosis* complex (Butler & Guthertz 2001). Such a diagnosis was reinforced by use of the normal-phase HPLC to separate the individual alpha-, methoxy- and keto-mycolates, followed by reverse-phase HPLC of each of these components, as demonstrated previously for modern (Minnikin *et al*. 1993) and ancient (Gernaey *et al*. 2001; Hershkovitz *et al*. 2008) tuberculosis. The final reverse-phase HPLC profiles were correlated with precise structural data for *M. tuberculosis* complex mycolic acids, recorded by Watanabe *et al*. (2002). Solvent blanks were run after any positive sample to ensure that there was no carry-over of material. The absolute amounts and percentage ratios of mycolic acid types were calculated as described previously (Hershkovitz *et al*. 2008).

## 3. Results

**Figure 2.**
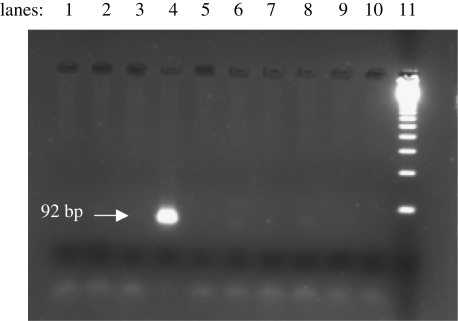
Demonstration of *M. tuberculosis* complex DNA in mummy lung sample. Lane 4: original lung sample showing a 92 bp nested PCR product specific for the *M. tuberculosis* complex; lanes 1–3 and 5–8: old lung extracts stored at −20°C and thawed; lanes 9 and 10: negative extraction controls; lane 11: 123 bp ladder.

Negative controls were satisfactory for both DNA and HPLC analyses. DNA from the *M. tuberculosis* complex was detected in the lung samples, gall bladder and membranous tissues (possibly pleura and diaphragm) but results were inconsistent (electronic supplementary material, table S2). Additional characterization of the DNA was not attempted as previously positive DNA extracts lost activity after freezing and thawing, indicating the fragility of the DNA. A positive result from the original lung tissue is shown on an agarose gel (figure 2). DNA sequences were obtained from the nested PCR products of the original lung sample, gall bladder and possible diaphragm, which were homologous with that in the NCBI database (electronic supplementary material, fig. S1*a*–*c*).

**Figure 3.**
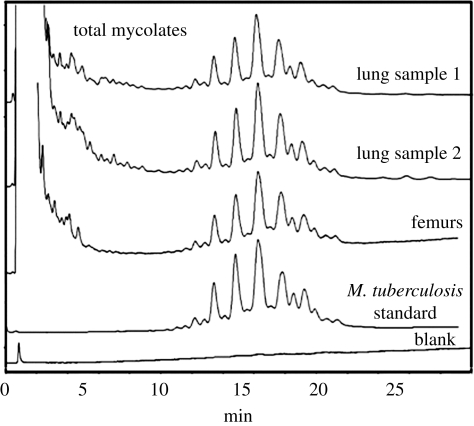
Detection of *M. tuberculosis* complex mycolic acid PBA–PFB derivatives by reverse-phase fluorescence HPLC, from the Granville mummy femur and lung samples, in comparison with a known standard strain of *M. tuberculosis*. The characteristic tight envelopes of peaks are the total mixture of homologues for the different alpha-, methoxy- and keto-mycolates. The *y*-axis in the profiles represents absorbance.

**Figure 4.**
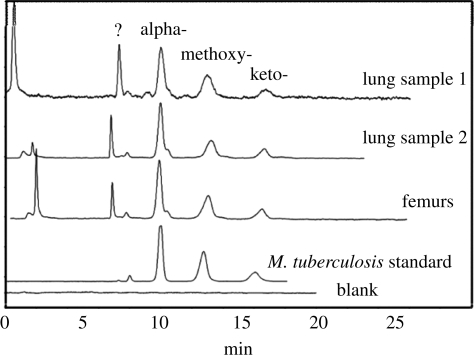
Normal-phase HPLC analyses of the collected total mycolates from the reverse-phase separation, from the Granville mummy femur and lung samples, in comparison with a known standard strain of *M. tuberculosis*. The *y*-axis in the profiles represents absorbance. The question mark indicates an unidentified peak.

**Figure 5.**
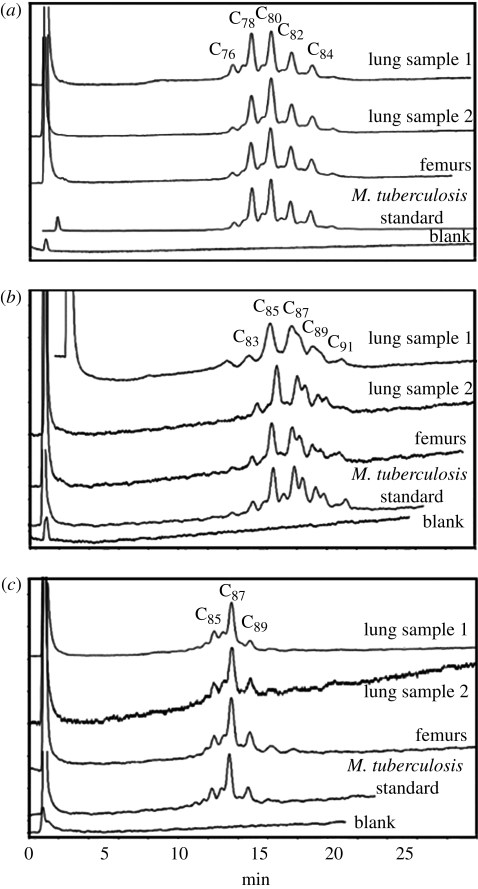
Reverse-phase HPLC analyses of the collected fractions from the normal-phase HPLC separation, from the Granville mummy femur and lung samples, in comparison with a known standard strain of *M. tuberculosis*. (*a*) Alpha-mycolates, (*b*) methoxy-mycolates and (*c*) keto-mycolates. The *y*-axis in the profiles represents absorbance.

The HPLC results for the analysis of extracts of combined left and right femurs and a new lung sample are shown in reverse-phase (figure 3), normal-phase (figure 4) and subsequent reverse-phase HPLC (figure 5). There was a good correlation between the profiles from each sample, with the *M. tuberculosis* positive standard, clearly demonstrating the presence of specific biomarkers for tuberculosis. The absolute amounts of mycolates in each sample were calculated (table 1). Lung sample 1 contained about twice the level of mycolates in sample 2 and a weaker signal was obtained from the combined femur samples. The proportions of alpha-, methoxy- and keto-mycolates (table 2) again correlated well with those from standard *M. tuberculosis* in the stronger lung sample 1, and the weaker samples followed the same trend.

**Table 1.** Absolute amounts of mycolates detected in lung samples and femurs from the Granville mummy.

mummy sample	sample mass (mg)	mycolate detected (µg)	mycolate/ sample mass (µg g^−1^)	ratio lung/femur
lung				
sample 1	29.6	0.397	13.41	3.3
sample 2	69.9	0.450	6.44	1.6
femurs	71.2	0.291	4.09	1

**Table 2.** Percentage ratio of individual mycolate types in lung samples and femurs from the Granville mummy.

	alpha-mycolate	methoxy-mycolate	keto-mycolate
lung sample 1	1	0.73	0.31
lung sample 2	1	0.51	0.25
femurs	1	0.67	0.25
*M. tuberculosis* standard	1	0.77	0.31

## 4. Discussion

The tissue samples used in these studies contained a relatively large quantity of what seemed to be insoluble foreign substances introduced during mummification. For example, the samples of original lung and gall bladder were noticeably oily. This mummy was unusual as it still had most of its internal organs *in situ* (Granville 1825). Therefore, the process of mummification appears to have differed from the norm. Granville believed that after initial dehydration with natron, the main material used was bitumen, possibly mixed with beeswax, but an analysis of embalming residues from the mummy has found no traces of either substance (Harer & Taylor in press). It is possible that the unusual process employed by the embalmers on this body has affected the retrieval of amplifiable DNA, as it has been readily obtained from other archaeological specimens from the Theban necropolis.

Tuberculosis was an endemic disease in ancient Egypt, and *M. tuberculosis* complex DNA has been found in the young and old, from high- and low-status burials. Where additional molecular characterization has been possible (Zink *et al*. 2001, 2003*a*,*b*), this has shown that human lineages of the *M. tuberculosis* complex were present: *M. tuberculosis* and possibly *Mycobacterium africanum*, but not *Mycobacterium bovis*. This is consistent with our current understanding of the evolution of the *M. tuberculosis* complex and the long-term coexistence of human populations with this microbial pathogen (Brosch *et al.* 2002; Hershkovitz *et al*. 2008; Wirth *et al*. 2008). In the present study, the *M. tuberculosis* complex was not further characterized owing to the difficulties described above. However, it is likely that the lady Irtyersenu was infected with a strain of *M. tuberculosis* of modern lineage, as these were prevalent in the populations examined by Zink *et al*. (2003*a*).

The detection of *M. tuberculosis* complex cell-wall mycolic acid biomarkers avoids the problems of DNA analysis. The molecules are far more robust and the method of analysis is sufficiently sensitive to permit the direct detection of femtogram quantities of material. Therefore, a direct biomarker analysis is an excellent alternative procedure for the confirmation of DNA findings. In addition, detection of lipid biomarkers appears to have potential for the examination of archaeological specimens that are too ancient or have been stored under suboptimal conditions, where DNA detection is not possible.

In the case of the Granville mummy, *M. tuberculosis* complex DNA was detected in the lung tissue, gall bladder and tissues from possible pleura and diaphragm. The HPLC data found the greatest quantity of *M. tuberculosis* complex cell-wall mycolates in the lung tissue and lower amounts in the femurs. Assuming that the pulmonary exudate was the prime source of the infection, it appears that the lady Irtyersenu had pulmonary tuberculosis that had disseminated to other sites in the body. During the second autopsy of Irtyersenu in 1994, it was noted that fat was interspersed in her skeletal muscle, which is indicative of wasting rather than obesity (Harer & Taylor in press). This suggests a somewhat protracted terminal illness and/or very sedentary lifestyle—both of which are consistent with an active tuberculosis infection. These molecular findings, combined with the histological observations, support a diagnosis of terminal disseminated pulmonary tuberculosis, rather than the malaria suggested by the second autopsy. Therefore, we are able to enhance the original paper by Granville (1825) to the Royal Society by concluding that there is evidence of an active tuberculosis infection in the lady Irtyersenu and that this, rather than a benign ovarian cystadenoma, was likely to be the major cause of her death.
